# Immune checkpoint inhibitor related nephrotoxicity: Advances in clinicopathologic features, noninvasive approaches, and therapeutic strategy and rechallenge

**DOI:** 10.3389/fneph.2022.1017921

**Published:** 2022-10-19

**Authors:** Jing Miao, Meghan E. Sise, Sandra M. Herrmann

**Affiliations:** ^1^ Division of Nephrology and Hypertension, Mayo Clinic, Rochester, MN, United States; ^2^ Department of Internal Medicine, Division of Nephrology, Massachusetts General Hospital, Boston, MA, United States

**Keywords:** immune checkpoint inhibitors, immune-related adverse events, nephrotoxicity, acute kidney injury, acute interstitial nephritis, liquid biopsy, noninvasive biomarkers, rechallenge

## Abstract

Immune checkpoint inhibitors (ICIs) are used increasingly to treat more than 17 cancers and have shown promising therapeutic results. However, ICI use can result in a variety of immune-related adverse events (IRAEs) which can occur in any organ, including the kidneys. Acute kidney injury (AKI) is the most common nephrotoxicity, classically related to acute interstitial nephritis. Much more diverse patterns and presentations of ICI-related kidney injury can occur, and have implications for diagnostic and therapeutic management approaches. In this review, we summarize the recently approved ICIs for cancer, the incidence and risk factors for nephrotoxicity, our current understanding of the pathophysiological mechanisms and the key clinicopathological features of ICI-related AKI, and therapeutic strategies. We also explore important knowledge that require further investigation, such as the risks/benefits of ICI rechallenge in patients who recover from an episode of ICI-related AKI, and the application of liquid biopsy and microbiome to identify noninvasive biomarkers to diagnose and predict kidney injury and guide ICI therapy.

## Introduction

Immune checkpoint inhibitors (ICIs) are monoclonal antibodies designed to interfere with surface receptors that mediate a negative feedback loop in the immune system. These receptors are mainly present on immune cells such as T and B cells, but are also expressed by certain tumor cells. By binding to these receptors, ICIs can inhibit and/or remove “the brakes” of the immune system. As a consequence, ICIs enhance activation of T cells and exert antitumor activity (1–3). Cytotoxic T lymphocyte–associated protein 4 (CTLA-4), also known as CD152, is a CD28 homolog that is predominantly expressed on T cells. It has a high binding activity for B7 (CD80/86) mainly present on antigen-presenting cells, and interaction of CTLA-4 and B7 can decrease and/or prevent T cell activation (4). Ipilimumab, a CTLA-4 inhibitor, was the first ICI drug approved by the U.S. Food and Drug Administration (FDA) for the treatment of melanoma in 2011 (5). Since the approval of ipilimumab, the list of the FDA-approved ICIs is increasing (**Table 1**); subsequently, six ICI inhibitors that target programmed cell death protein 1 (PD-1) (cemiplimab, nivolumab, pembrolizumab), or PD-ligand 1 (PD-L1) (atezolizumab, avelumab, durvalumab) have been approved by FDA (1, 6, 7). PD-1 is a receptor mainly localized on the plasma membrane of T cells, while PD-L1 is expressed on the surface of tumor and antigen-presenting cells. PD-1 and PD-L1 interaction can inhibit activation of T cells, thus maintain a balance of immune homeostasis and result in blockade of anti-tumor immune response (8–10). Recently, based on data from a global, randomized phase 2-3 study, RELATIVITY-047 (11), the FDA approved relatlimab, the first immunotherapy that targets the lymphocyte activation gene-3 (LAG-3) for patients aged 12 and older with untreated metastatic or unresectable melanoma, combined with the PD-1 inhibitor nivolumab. The indications for ICIs have also been expanded to many types of malignancies (i.e., melanoma, renal cell carcinoma, non-small cell lung carcinoma, metastatic colorectal cancer, breast, and classic Hodgkin lymphoma) (**Table 1**) (12).

**Table 1:** Food and drug administration-approved immune checkpoint inhibitors.

Drug	Initial approval (year)	Target	Indication
Ipilimumab	2011	CTLA-4	Melanoma, renal cell carcinoma, MSI-H or dMMR metastatic colorectal cancer, non-small cell lung cancer, malignant pleural mesothelioma
Cemiplimab	2018	PD-1	Cutaneous squamous cell cancer, basal cell carcinoma, non-small cell lung cancer
Nivolumab	2014	PD-1	Melanoma, non-small cell lung cancer, malignant pleural mesothelioma, renal cell carcinoma, classic Hodgkin’s lymphoma, head and neck squamous-cell carcinoma, urothelial carcinoma, MSI-H or dMMR metastatic colorectal, hepatocellular carcinoma, esophageal cancer, gastric cancer, gastroesophageal junction cancer, and esophageal adenocarcinoma
Pembrolizumab	2014	PD-1	Melanoma, non-small cell lung cancer, head and neck squamous-cell carcinoma, classic Hodgkin’s lymphoma, primary mediastinal large B cell lymphoma, urothelial carcinoma, MSI-H or dMMR solid tumors, MSI-H or dMMR colorectal cancer, gastric cancer, esophageal cancer, cervical cancer, hepatocellular carcinoma, Merkel cell carcinoma, renal cell carcinoma, endometrial carcinoma, tumor mutational burden-high cancer, cutaneous squamous cell carcinoma, triple-negative breast cancer
Atezolizumab	2016	PD-L1	Urothelial carcinoma, non-small cell lung cancer, small cell lung cancer, hepatocellular carcinoma, melanoma
Avelumab	2017	PD-L1	Merkel cell carcinoma, urothelial carcinoma, renal cell carcinoma
Durvalumab	2017	PD-L1	Urothelial carcinoma, unresectable Stage III non-small cell lung cancer, extensive-stage small cell lung cancer (in combination with etoposide and either carboplatin or cisplatin)
Relatlimab	2022	LAG-3	Untreated metastatic or unresectable melanoma (in combination with nivolumab)

CTLA-4, cytotoxic T-lymphocyte antigen 4; dMMR, mismatch repair deficient; MSI-H, microsatellite instability-high; PD-1, programmed cell death 1; PD-L1, programmed cell death ligand 1; LAG-3, lymphocyte activation gene-3.

The success of ICIs has come at the cost of a distinct spectrum of side effects known as immune-related adverse events (IRAEs). IRAEs can occur in any organ in patients receiving ICIs, with a wide range in incidence from 59% to 85% overall (13, 14). IRAEs present with various degrees of severity from mild to life-threatening. The organs most commonly affected by IRAEs are the skin, gastrointestinal tract, and endocrine system; the kidneys are less frequently affected (15). Acute kidney injury (AKI) caused by acute interstitial nephritis (AIN) is the major type of ICI-related kidney injury or nephrotoxicity (6), but increasing evidence has also demonstrated other patterns of ICI-related nephrotoxicity, such as glomerular diseases (e.g., pauci-immune glomerulonephritis, C3 glomerulonephritis, renal vasculitis, and podocytopathy), acute tubular necrosis (ATN), and electrolyte abnormalities (e.g., hyponatremia, hypocalcemia, renal tubular acidosis [RTA]). Due to different mechanisms of action among ICI classes, the clinicopathological presentations might vary among patients treated with different ICIs. ICI-related kidney IRAEs pose unique diagnostic and management challenges.

In the current review, we discuss our understanding of the incidence, pathophysiological mechanisms, risk factors, key clinicopathological features, and therapeutic strategies and outcomes of ICI-related nephrotoxicity. We also discuss on-going studies and the gaps in knowledge that require further investigation, such as ICI rechallenge in patients whose kidney function recovered from an episode of ICI-related AKI, and the application of liquid biopsy and microbiome to identify noninvasive biomarkers for diagnosis and prediction of kidney injury as well as guidance of ICI therapy.

## Epidemiology of ICI-related AKI

Precise data on the incidence of ICI-related adverse kidney effects such as AKI remain limited as large population-based studies across diverse international cohorts are not available. Available data, however, indicate that the incidence of AKI (defined by at least 1.5 times increase in serum creatinine relative to the baseline) in patients receiving ICI is approximately 17% (16–20). Early cohort studies reported an estimated incidence of AKI from 10% to 30% (21), but most recent studies noted a much smaller risk in the order of 3 to 5% (16, 22–25). In agreement, a systematic meta-analysis study including 48 clinical trials and 11,482 patients treated with PD-1 inhibitors demonstrated that the overall incidence of AKI was 4.2% (26). Furthermore, a retrospective study in 1766 patients receiving an ICI between 2014 and 2018 found that 7.6% (n=123) developed AKI within one year of administration of the first dose, but the incidence of AKI directly related with ICI use was only 0.8% (27). While most studies support that there is no difference in AKI risk with CTLA-4 and PD-1 inhibitor monotherapy (16–18, 22–26, 28, 29), PD-L1 inhibitors such as atezolizumab, durvalumab and avelumab may have a lower risk of AKI (<1%) (30). However, combination of the CTLA-4 inhibitor ipilimumab and the PD-1 inhibitor nivolumab carries a higher risk of ICI-related AKI (4.9%) than monotherapy with either ipilimumab (2%), nivolumab (1.9%), or pembrolizumab (1.4%) (28). Data from some clinical trials also shows less organ-toxicity such as pneumonitis and colitis in patients receiving PD-L1 inhibitors than PD-1 and CTLA-4 inhibitors (6, 31, 32). However, post-marketing surveillance data indicate that atezolizumab may have the highest risk of kidney side effects among all ICI monotherapies but confirm a low risk of ICI-related AKI with avelumab (33). So far, data on the “real-world” incidence of LAG-3 inhibitor-related AKI or other kidney side effects are unavailable. In the global phase 2-3 RELATIVITY-047 trial the incidence of ICI-related kidney adverse effects, such as nephritis and renal dysfunction that occurred within 100 days after the last dose, was only 2% in patients receiving combination therapy of relatlimab and nivolumab, compared to 1.4% in those receiving nivolumab alone (11).

In summary, the incidence of AKI directly related to ICI is low, but ranges from 0.8% to 5%. This is likely due to a variety of factors, such as patients’ demographics, definitions used to categorize AKI, different half-life of ICIs, single or combination use of ICIs, and cancer types. The epidemiology and/or incidence of ICI-related AKI and other kidney side effects, particularly in those patients with the single ICI use need be further investigated and validated by large, prospective, multicenter cohort studies.

## Clinical features of ICI-related nephrotoxicity

AKI is common in patients with cancer, but ICI-related AKI that is directly caused by the use of ICI, not by other alternative etiologies, is less common. Unfortunately, clinical characteristics that can be used to distinguish ICI-related AKI from other causes-related AKI remain unavailable or unreliable, although there are some features suggestive of ICI-related AKI. A multicenter study of 138 patients with ICI-related AKI (defined by ≥2 times increase in serum creatinine or new dialysis requirement) found that most patients had sub-nephrotic proteinuria, about half presented with sterile pyuria, and a small portion of patients (~21%) had eosinophilia (24), similar to the findings from other studies (25, 28, 34, 35). Nevertheless, these clinical manifestations are not specific enough to confirm or rule out ICI-related AKI because other causes such as drug-related AKI have similar features.

A multicenter study shows that approximately half of patients with ICI-related AKI had an extrarenal IRAE (i.e., colitis, dermatitis, hypophysitis, rash, or thyroiditis) that occurred before or at the time of AKI, and rash was considered as the most common manifestation of extrarenal IRAEs (24). Studies by Seethapathy et al. and Cortazar et al. showed that the incidence of ≥1 extrarenal IRAEs in patients with ICI-related AKI was up to 62% and 87%, respectively (16, 28), and the most common coexisting IRAEs were thyroiditis and colitis (16). Thus, the presence of extrarenal IRAEs may be a crucial clinical clue when ICI-related AKI is suspected. In addition, it should be noted that AKI following ICI initiation often occurs later than other commonly reported IRAEs (36–38). As outlined in a multicenter study, the median interval from the initiation of ICI therapy to the occurrence of AKI was 14 weeks with an interquartile (IQR) range of 6-47 weeks (24). This is similar to the findings (median interval: 13 and 14 weeks, IQR range: 3-35 and 6-56 weeks, respectively) of two small studies with 13 and 16 patients, respectively (25, 28). Of note, ICI-related AKI may occur at any time after initiation of ICI therapy, from a few days after the first dose of ICI use to more than 10 weeks after the last dose given (24). Importantly, however, as shown in our studies, ICI-related AKI occurred earlier than AKI due to other causes in cancer patients undergoing ICI therapy (median [IQR], 4 months [1.2, 11.4] vs. 8.5 months [5.3, 10.4], *p = 0.03*), and use of proton pump inhibitors (PPI) or other AIN-associated drugs led to more rapid onset of AKI (22). Therefore, the variation of the latency period from ICI initiation to AKI occurrence is large, probably caused by long, different half-lives (6-27 days) of ICI therapy (39). Additional data are needed to clarify the interval between ICIs administration and AKI development.

Although ICI-related AKI is the most common kidney IRAE, ICI-related electrolyte abnormalities have also been described, such as hyponatremia, hypokalemia, hyperkalemia, hypophosphatemia, and hypomagnesemia (14, 21, 40–47). A narrative review covering all ICI-related renal toxicities that have been reported to the FDA adverse event reporting system between July 2011 and June 2015, confirmed that hyponatremia was the second most common type of ICI-related renal toxicities, accounting for 33.9% of reports (21). Hyponatremia was more common with the CTLA-4 inhibitor ipilimumab than the PD-1 inhibitors nivolumab and pembrolizumab (21). It needs to be recognized though that hyponatremia in general is not uncommon in patients with cancer (up to 50%), likely due to volume depletion, cirrhosis, and syndrome of inappropriate anti-diuretic hormone (48–50). As ICI can cause autoimmune adrenalitis or hypophysitis (7), ICI hyponatremia may also be caused by primary or secondary adrenal insufficiency (7). Severe hyponatremia (<124 mEq/L) due to ICI-related endocrinopathy, however, is rare, affecting only 0.3% of patients treated with ICIs (51). Regarding hypercalcemia, a systematic meta-analysis study including 48 clinical trials with 11,482 patients shows that the overall incidence in patients who received a PD-1 inhibitor is 1% (95% confidential interval (CI) 0.6-1.8%) (26). Severe hypocalcemia defined by a corrected calcium <7.0 mg/dL is a rare event, estimated to occur in only 0.2% in patients receiving ICIs (51).

Cases of distal RTA have been described in patients receiving ICIs who develop AIN. In 2018, the first presentation of RTA as a renal IRAE was reported in a 79-year-old female with metastatic non-small cell lung cancer following treatment with the PD-1 inhibitor nivolumab (52). In a 59-year-old male patient with a stage IV bronchopulmonary cancer, tubular dysfunction developed and preceded AKI following initiation of the PD-1 inhibitors nivolumab and pembrolizumab (53). It has also been shown that distal RTA persisted in some cases with ICIs therapy although kidney function had already improved (54, 55). A study analyzing kidney biopsies by immunofluorescent staining with specific antibodies found that expression of type 1 and B1 anion exchanger and vacuolar-type H^+^-ATPase α4 subunit was decreased in the intercalated cells in comparison with controls (54). This suggests at least partially that in the context of ICI-related autoimmune response, expression of H^+^-ATPase and/or Cl^-^/HCO3^-^ was altered in the type A intercalated cells.

Finally, ICIs can also reactivate pre-existing autoimmune diseases (56). It is estimated that 27-50% of patients with preexisting autoimmune disease (i.e., inflammatory bowel disease, lupus, and rheumatoid arthritis), experienced worsening of their disease following treatment with an ICI (57). Based on our knowledge, there is only one case report of ICI-related primary membranous nephropathy reactivation. In this case with pleural mesothelioma, primary membranous nephropathy was reactivated following nivolumab treatment, and rituximab, specifically targeting B-lymphocytes, was effective in treating reoccurrence of membranous nephropathy. The patient was rechallenged with nivolumab and kidney function remained stable (58). There is also a case of a 69-year-old patient with metastatic melanoma who had pre-existing anti-neutrophil cytoplasmic autoantibody (ANCA) vasculitis and ipilimumab-related colitis. In this case, his vasculitis did not flare following PD-1 inhibitor pembrolizumab therapy (59).

## Pathological features of ICI-related nephrotoxicity

Many studies have demonstrated that the most common pathologic lesion of ICI-related AKI is AIN, which was reported in nearly 90% of kidney biopsies (24, 25, 28, 34). However, other pathologic entities that occur either alone or in conjunction with AIN also have been described, including various glomerulonephritis and acute tubular injury or ATN (16, 18, 25, 28, 60, 61). The incidence of these glomerular diseases remains to be defined; but our estimate is that these pathologic lesions account for <10% of all cases of ICI-related AKI.

Glomerular disease is less common than AIN, but remains a crucial adverse effect of ICIs. In a large, multicenter series study, AIN was reported in 93% (n=56/60) of patients with ICI-related AKI who underwent biopsy. The remaining patients were diagnosed with minimal change disease with acute tubular injury, ANCA-negative pauci-immune crescentic glomerulonephritis, anti-glomerular basement membrane disease, and C3 glomerulonephritis (24). Another international multicenter study that included > 400 cases of ICI-related AKI showed that among patients who underwent kidney biopsy, the final diagnosis was AIN, ATN, IgA and membranous nephropathy for 82.7%, 6.3%, 2.5% and 2% of the cases, respectively (35). Smaller series in patients with advanced solid or hematologic malignancy who developed ICI-related AKI indicated the same: 88% (14 out of 16 patients) presented with AIN on kidney biopsy either as the major pathologic form or as the mild interstitial inflammation associated with other glomerular diseases (i.e., membranous glomerulonephritis, pauci-immune glomerulonephritis, C3 glomerulonephritis, IgA nephropathy, and amyloid A amyloidosis) (25). Minimal change disease was described in one patient receiving the anti-PD-1 antibody pembrolizumab for Hodgkin lymphoma and two patients receiving the CTLA-4 antibody ipilimumab for melanoma, respectively (62, 63). In addition, focal segmental glomerulosclerosis, lupus nephritis, and renal vasculitis after ICIs have been reported in several case reports (25, 60, 64, 65). A case series reported that renal vasculitis or pauci-immune glomerulonephritis occurred in four patients after ICI therapy. Interestingly, three patients presented with small to medium vessel vasculitis, but had negative ANCA testing (60). A recent study of 45 patients with biopsy-proven glomerular disease following ICIs therapy suggested that the most common kidney lesions were pauci-immune glomerulonephritis/vasculitis (n=12/45, 27%), podocytopathy (n=9/45, 20%), and C3 glomerulopathy (n=5/45, 11%) (61). Similar to ICI-related AIN, the median time from the ICI initiation to the diagnosis of glomerular disease was about 3 months. It should be noted that among patients with glomerular diseases, 40% also had AIN on biopsy (61). All of the 12 patients with pauci-immune glomerulonephritis had negative ANCA testing results, and of note, and none were taking medications such as hydralazine and/or minocycline that are typically associated with occurrence of ANCA vasculitis (61).

Finally, ATN is a noteworthy pathological feature associated found in kidney biopsies of patients who develop AKI while on ICI therapy. However, it is unclear if ATN is truly an IRAE. A small case series of 12 patients who received pembrolizumab therapy showed 5 cases with ATN, 4 with AIN and 2 with minimal change disease (66). In another series of 15 patients with AKI following ICI therapy and renal biopsy results, 9 patients had AIN and 6 had ATN (67).

In summary, the histopathological patterns of kidney injury in patients with ICIs therapy are largely heterogenous. Therefore, kidney biopsy is very important in those patients with a clinical history that highly suggests ICI-related AKI.

## Risk factor of ICI-related nephrotoxicity

Several risk factors have been reported to be related to ICI-related AKI. Particularly PPI use, which is also associated to AIN in the general population, is a significant risk factor for ICI-related AKI (68). Two large retrospective studies show that the PPI use was significantly associated with ICI-related AKI, and both studies show that the hazard ratio was 2.85 (95% CI 1.81 and 4.48 to 1.34 and 6.08, respectively) (16, 24). Other drugs that have been reported to increase the risk of ICI-related AKI include non-steroidal anti-inflammatory drugs (NSAIDs) and antibiotics (16, 24). Current hypotheses suggest that administration of drugs that potentially increase the risk of AIN may initiate the immune reaction triggered by drug-specific T cells (69). These latent drug-specific T cells may be reactivated following ICIs therapy, leading to loss of immune tolerance (34, 70). As such, drugs known to cause or increase the risk of AIN should be used cautiously in patients who are receiving ICIs therapy. Interestingly, a large, multicenter cohort study shows that patients with ICI-related AKI who received AIN-associated drugs more likely recovered from AKI, possibly due to discontinuation of these nephrotoxic medications (24, 35). Other studies also show that additional risks for ICI-related AKI included lower estimated glomerular function rate at baseline, combination therapy of ICIs, and presence of extrarenal IRAEs (16, 24, 35).

## Pathophysiology: the underlying mechanisms of ICI-related kidney injury

**Figure 1:**
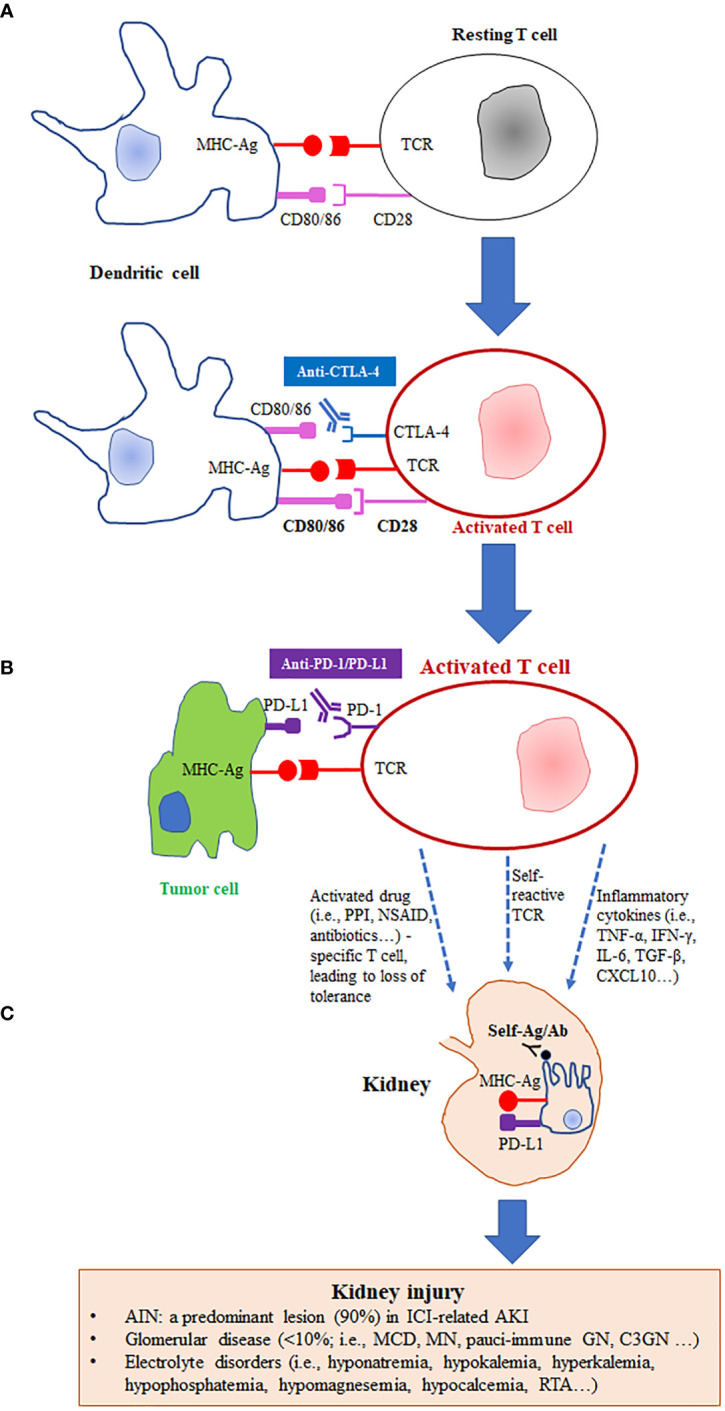
Proposed mechanisms of immune checkpoint inhibitor-related kidney injury. **(A)** CTLA-4 competes with CD28 receptor binding to CD80/CD86 on dendritic cells, leading to inhibition of T cell activation and thereby avoiding autoimmune response. Anti-CTLA-4 (i.e., ipilimumab) blocks CD80/CD86 binding to the CTLA-4 receptor, which allows CD28 binding and induces activation of T cells. **(B)** PD-L1, the ligand of PD-1, is expressed on certain tumor cells. PD-L1 binds PD-1 and blunt T cell function. Blocking PD-L1 binding to PD-1 with anti-PD-1 or anti-PD-L1 monoclonal antibodies (i.e., nivolumab and atezolizumab) further activates T cells or suppresses deactivation of T cells and promotes tumor cell killing. **(C)** Activated T cells increase proinflammatory cytokines/chemokines in kidney tissue and generate autoantibodies against kidney tissue. Reactivation of drug specific effector T cells by ICIs leads to loss of tolerance. The elevation of PD-L1 on renal tubular epithelial cells can cause kidney injury through locally infiltrated effector T cells. CTLA-4, cytotoxic T lymphocyte–associated protein 4; PD-1, programmed cell death protein 1; PD-L1, programmed death ligand 1; MHC-Ag: major histocompatibility complex with tumor antigen; TCR, T cell receptor; PPI, proton pump inhibitors; NSAID, non-steroidal anti-inflammatory drug; AIN, acute interstitial nephritis; ICIs: immune checkpoint inhibitors; AKI, acute kidney injury; MCD, minimal change disease; MN, membranous nephropathy; GN, glomerulonephritis; RTA, renal tubular acidosis.

To date, the precise mechanisms of ICI-related AKI have not been well defined. Immune checkpoints play a key role in maintaining normal immune responses and self-tolerance. After activation, PD-1 expression is increased on T, B, and natural killer T cells, as well as the activated monocytes and dendritic cells (71). Its ligand PD-L1 is localized on the membrane of tumor cells (72). The PD-1 and PD-L1 interaction results in blockade of anti-tumor immune responses (8–10). CTLA-4, specifically expressed on the surface of T cells, can prevent or decrease immune response by competing the binding of CD28 to CD80 or CD86 on dendritic cells (73). Furthermore, CTLA-4 can regulate the interactions between T and the antigen-presenting cells in the secondary lymphedema (74). As a result, the ICI-induced blockade of the PD-1/PD-L1 and CTLA-4 axis can cause activation of T cells in tumor tissue, which then kill tumor cells by producing inflammatory factors and cytokines (**Figure 1**).

However, the initiation of this blockade may result in release of tissue-specific self-reactive T cells, which can reduce or disturb immune tolerance against renal antigens. When circulate into kidney, the aberrant activated T lymphocytes infiltrate into the parenchyma and release cytokines, leading to inflammatory reaction and kidney injury (75–77) (**Figure 1**). In a preclinical study, a chronic systemic inflammation and a lupus glomerulonephritis-like kidney lesion developed in mice with PD-1 knockout mice (78), providing evidence that loss or inhibition of PD-1 has kidney side effects. In addition, due to changing the immune microenvironment, exposure of ICIs may produce drug-specific antibodies against renal tissue or circulating antibodies against native antigens (79). This may lead to immune complex-related AIN. Exposure of ICIs may reactivate drug-specific T cells and then decrease their tolerance to AIN-associated drugs. The reduction of tolerance may increase the likelihood of nephrotoxicity in patients with combination therapies of ICIs and PPI or NSAIDs.

Haptenization may be another potential mechanism of ICI-related AKI. Small molecular weight drug compounds such as ICIs or their metabolized components can be recognized as an antigen by mesangial cells, kidney tubular cells, or glomerular podocytes, leading to formation of antigen-antibody complex (80). Also, ICIs can be trapped and recognized as a hapten by dendric cells in the parenchyma of kidney, leading to tubular damage and kidney injury (81).

In addition, PD-L1 is expressed both on the membrane of tumor cells and normal kidney tubule, especially the proximal tubular cells (82). In a murine model of nephrotoxic nephritis, infiltration of Foxp3^+^ Tregs expressing PD-L1 was detected in the kidneys (83). Inhibition of PD-L1 signaling by PD-L1 knockout or by anti- PD-L1 antibody increased Treg cells in the inflamed kidney, but more severe kidney injury was observed (83). This is likely associated with increase of kidney Th1 immune response. Notably, the gene expression profile of Tregs in kidney inflammation was significantly changed in PD-L1 knockout mice (83). Moreover, the inhibitory ability of Tregs isolated from kidney PD-L1 knockout mice were impaired *in vitro*. These Tregs did not protect against ATN *in vivo* (83). In a kidney ischemia-reperfusion injury model, administration of anti-PD-L1 or anti-PD-L2 antibodies significantly abrogated Treg-mediated protection function and exacerbated kidney inflammation and tubular injury (84). These data suggests that PD-L1 plays a protective role in kidney injury, which might be associated with Treg-mediated inhibition of the Th1 immune response. Just recently, acute rejection was observed in kidney transplant mice that received anti-PD-1 and anti-PD-L1 antibodies. Interestingly, increased infiltration of T-cells and expression of urinary lipocalin 2 were detected in these mice (85). Clinically, increased PD-L1 expression was reported in patients who underwent rejection after a kidney transplant (86); this might be a compensative upregulation. These findings suggest that PD-L1 inhibitors may execute different effects through regulating function of multiple types of cells in kidney injury (87).

## Imaging and diagnostic tools of ICI-related nephrotoxicity

Generally, patients receiving ICI therapy have routine laboratory tests such as complete blood count and comprehensive metabolic panel before initiation of ICIs; urinalysis is not currently recommended, but should be considered as a part of the initial baseline tests in patients at risk for kidney disease. As such, physicians may have more baseline information about kidney function for comparison during ICIs therapy. The following tests should be considered to improve clinical diagnosis, prognosis, and therapeutic efficacy in patients who develop ICI-related kidney injury.

### Kidney biopsy

The gold standard for diagnosis of ICI-related AIN or glomerular disease is kidney biopsy. First, alternative etiologies for AKI should be excluded, such as contrast-related nephropathy, volume depletion, obstructive nephropathy, and chemotherapy related renal tubular injury. Current guidelines recommend that empiric immunosuppressive therapy of suspected ICI-related AIN is acceptable without a kidney biopsy if other causes and glomerular disease can be excluded, unless there are moderate or severe life-threatening kidney side effects defined as Grade 2 to 3 or higher by Common Terminology Criteria for Adverse Events (CTCAE) (88, 89). However, this poses a potential risk to patients if unnecessary immunosuppression is initiated. Therefore, besides a complete and thorough kidney function assessment, kidney biopsy is often required to diagnose the precise kidney lesion and provide optimal therapy (7). Of note, additional histological stains may be helpful to in the diagnosis of ICI-related AKI. A study shows that positive PD-L1 staining in tubular epithelial cells can help differentiate PD-1-related AIN from other causes-related AIN (67). Nevertheless, further studies are still needed to validate the diagnostic role of PD-L1 staining in PD-1-related AIN (6).

### Imaging studies

Noninvasive tests are needed to diagnosis AIN in patients who cannot undergo kidney biopsy; however, so far, their diagnostic utility is limited. A few reports have shown that uptake of ^18^F-fluorodeoxyglucose (FDG) on positron emission tomography (PET) scan can be increased in renal cortex in patients with ICI-related AIN (90, 91). However, patients with ICI-related AKI caused by other non-AIN causes can also have mild FDG uptake (90, 92). As such, PET scan may not be as diagnostic in patients with mild AIN, especially in those who do not have a baseline PET scan image for comparison. But for those patients who cannot undergo kidney biopsy, PET scan may be used as an adjuvant diagnostic tool for ICI-related AIN when other causes-related AKI are excluded (7).

### Liquid biopsy (noninvasive biomarkers)

Kidney biopsy is the gold standard for diagnosis of ICI-related AKI providing guidance for therapeutic decisions. However, its big limitation is the invasive nature of the procedure. Furthermore, kidney biopsy cannot be repeated multiple times. Based on a small tissue, the information derived from the biopsy samples are likely incomplete and unable to reveal the whole kidney histopathology. To overcome these challenges, minimally-invasive liquid biopsy of blood or urine is an attractive alternative for identification of biomarkers (93).

### Serum markers

It has been well known that systemic inflammatory status correlates with the occurrence of AKI. Some inflammatory factors, such as serum C-reactive protein (CRP), IL-6, IL-10, IL-17, TGF-β1, lactate dehydrogenase (LDH), lymphocyte number, and the ratio of neutrophil relative to lymphocyte, may be potentially used as the biomarkers for IRAEs. A study shows that the gene expression profile of peripheral blood before ipilimumab treatment was changed and associated with the occurrence of gastrointestinal IRAEs in patients with advanced melanoma (94). Subsequently, it has been reported that baseline serum IL-17 level was significantly correlated with severe diarrhea and colitis, while the combination use of baseline TGF-β1 and IL-10 levels was significantly (hazard ratio 2.66, *p = 0.035*) associated with therapeutic clinical outcome such as progression free survival after ipilimumab (95). In a phase II clinical trial including 27 patients with metastatic prostate cancer receiving ipilimumab, CD8^+^ T cell clones was found to be increased, which preceded the development of severe IRAEs such as rash, hypophysitis and diarrhea (96). Later on, some studies suggested that the baseline derived neutrophils/(leukocytes minus neutrophils) ratio and LDH level could be used as prognostic biomarkers in patients with advanced or metastatic non-small cell lung cancer (97, 98). It has been reported that the decreased ratios of neutrophil or platelet relative to lymphocyte can predict IRAEs (i.e., pneumonitis, diarrhea, increase lipase/amylase, increase transaminase, skin-related events, and arthralgies/myalgies) in patients with advanced non-small cell lung cancer (99). The levels of CRP, a type of acute phase protein, was reported to be increased prior to the occurrence of IRAEs in patients with ICIs therapy (100). Additionally, early increased CRP and IL-6 after the administration of nivolumab or pembrolizumab is also an indicator of cancer response in patients with non-small cell lung cancer (101). Thereafter, it has been shown that CRP, IL-6, and the neutrophil-to-lymphocyte ratio were negatively correlated with the overall survival in patients with metastatic melanoma who received nivolumab or ipilimumab, suggesting that they could be used as the prognostic markers (102). Interestingly, a recent study found that the levels of serum CRP and the ratio of urine retinol binding protein (URBP) relative to creatinine were significantly higher in patients with ICI-related AKI compared to those with non-ICI-related AKI (22). Additionally, a multivariate analysis of 38 patients with advanced non-small cell lung cancer with 32 paired serum samples revealed that follistatin (also known as activin-binding protein) and IFN-γ-inducible protein-10 (IP-10, also known as CXCL10) were statistically associated with durable clinic benefit defined by partial response and stable disease that lasted for more than 6 months while CCL5 (RANTES) was significantly associated with IRAEs onset. Reduction of CCL5 levels was also detected after initiation of corticosteroid treatment (103). Furthermore, a study shows that baseline level of CD16^+^ monocytes were positively associated with therapeutic response rate, but negatively correlated with Treg cell number in patients with ipilimumab therapy (104). Recently, a study including 1746 patients with metastatic breast cancer receiving ICIs therapy shows that increased infiltrations of lymphocytes and CD8^+^ T cells in tumor tissue can predict progression free survival and the overall survival (105).

### Urinary markers

It has been reported that urine TNF-α and IL-9 levels were persistently elevated in patients with AIN compared to those with other diagnoses (i.e., acute tubular injury, glomerular diseases, and diabetic kidney disease) and those without any known kidney diseases (106). This suggests that currently available tests of urinary TNF-α and IL-9 can be used to improve discrimination for AIN diagnosis before kidney biopsy. However, these studies included only a few patients treated with ICIs. A recent study including 37 patients with ICI-related AKI and 13 with non-ICI-related AKI found that at the time of AKI diagnosis, the ratio of URBP relative to urine creatinine was significantly higher (median [IQR] 1927 [1174, 46,522] vs. 233 [127, 989]) in patients with ICI-related AKI compared to those with the non-ICI-related AKI (22).

As described above, some serum and urine markers have shown promise for diagnosing IRAEs including AIN in a small series of patients (**Table 2**). Notably, the application of only one biomarker can be nonspecific for individual IRAEs. But multi-positive biomarkers can highly increase the sensitivity of diagnosis of renal IRAEs. Isik et al., showed that both the log (CRP & URBP/creatinine) is higher in patients with presumed ICI-AKI; therefore, helping with the decision on performing the kidney biopsy, particularly when other infectious diseases and other obvious causes are ruled out (22). Conversely, when both biomarkers are negative and patients are not already on any immunosuppression, the presence of ICI-related AKI is less likely (22). However, further validation is still needed in large, multicenter, prospective studies. In addition, the study of urinary proteomics may identify more potential biomarkers for kidney disease (93). Prospective studies that better defined the mechanisms of IRAEs may also identify more specific noninvasive biomarker candidates for ICI-related AKI.

**Table 2:** Potential noninvasive biomarkers for IRAEs and clinical outcomes in patients on ICI therapy.

Non-invasive Biomarkers	IRAEs	Clinical outcomes	Reference
Blood/serum			
IL-17	Baseline level correlated with severe diarrhea and colitis		(95)
CD8^+^ T	Increased prior to severe IRAEs (i.e., diarrhea, hypophysitis and rash)		(96)
CCL5	IRAEs (The detail was not mentioned in the study)		(103)
IL-10		In combination with IL-17, and TGF-β1, baseline levels associated with progression free survival	(95)
dNLR and LDH		Baseline levels correlated with worse outcomes (i.e., disease control rates, progression free survival, and the overall survival)	(97, 98)
IL-6		Associated with the therapeutic response, but negatively associated with the overall survival	(101, 102)
Follistatin and IP-10		Associated with the durable clinic benefit (partial response and stable disease that lasted for more than 6 months)	(103)
CD16^+^ monocytes		Baseline level associated with the therapeutic response	(104)
NLR and PLR	Low baseline associated with onset of IRAEs (i.e., pneumonitis, diarrhea, increase lipase/amylase and transaminase, skin-related events, and arthralgies/myalgies)	NLR: negatively associated with the overall survival	(99, 102)
CRP	Prognostic for IRAEs (i.e., ICI-related AKI)	Associated with the therapeutic response, but negatively associated with the overall survival	(22, 100–102)
Urine			
TNF-α and IL-9	Increased in AIN		(105)
URBP to UCr ratio	Increased in ICI-related AKI	Normal values less likely renal IRAEs	(22)

IRAEs, immune-related adverse events; ICI, immune checkpoint inhibitor; dNLR, derived neutrophils/(leukocytes minus neutrophils) ratio; LDH, lactate dehydrogenase; IP-10, IFN-γ-inducible protein-10; NLR, neutrophil-to-lymphocyte ratio; PLR, platelet-to-lymphocyte ratio; CRP, C-reactive protein; AKI, acute kidney injury; TNF-α, tumor necrosis factor alpha; AIN, acute interstitial nephritis; URBP, urine retinol binding protein; UCr, urine creatinine.

### Microbiome Gut

Microbiota regulates multiple physiological processes, and have an important role in various pathologic conditions, including obesity, diabetes, inflammatory bowel disease, neurodegenerative disease, and cancer (107). It has been reported that the gut microbiota may have a critical role in chronic kidney disease and AKI; but the current results are inconsistent (108, 109). Recently, a study found that the gut microbiota involved immune system regulation and tumorigenesis, and its abundance and composition influenced the efficacy of ICIs treatment (107). In addition, it has been reported that certain toxicities produced following ICI therapy were associated with the intestinal microbiome, particularly in patients with colitis (110). A study of patients with melanoma found that incidence of colitis was lower in those with rich *Bacteroidetes* following the treatment of anti-CTLA-4 antibody ipilimumab (111). However, the role of altered microbiome in ICI-related AKI remains unstudied, and the further investigation is needed.

## Management and outcomes of ICI-related nephrotoxicity

The severity of ICI-related AKI varies. It has been indicated that ICI-related AKI was not related to increased mortality (112); however, non-recovery of AKI has been shown to be a significant predictor for mortality (24). Therefore, early identification and prompt treatment appears to be important for management of IRAEs including ICI-related AKI.

### Suggested general management for patients with ICI-related AKI

**Figure 2:**
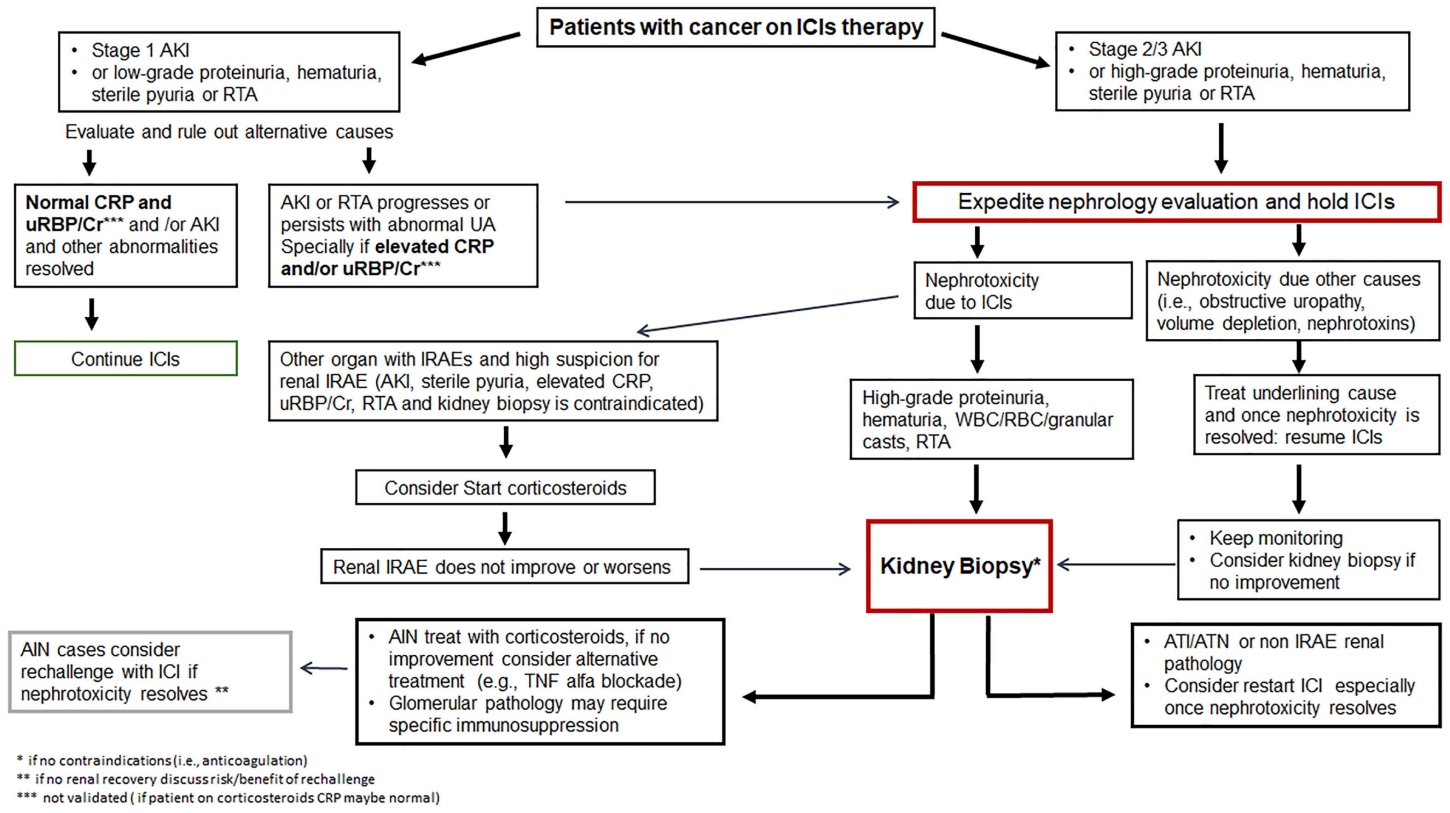
Management of immune checkpoint inhibitor related nephrotoxicity. AIN, acute interstitial nephritis; AKI, acute kidney injury; ATI, acute tubular injury; ATN, acute tubular necrosis; Cr, creatinine; CRP, C-reactive protein; GN, glomerulonephritis; ICIs, immune checkpoint inhibitors; IRAE, immune-related adverse event; RBC, red blood cell; RTA, renal tubular acidosis; UA, urinalysis; uRBP, urine retinol-binding protein; WBC, white blood cell.

Currently, the CTCAE grade ≥ 2 kidney toxicity (grade 2: 2-3x baseline creatinine; grade 3: >3x baseline creatinine; grade 4: >6x baseline creatinine or dialysis needed), the ASCO (American Society of Clinical Oncology), NCCN (National Comprehensive Cancer Network) and SITC (Society for Immunotherapy of Cancer) guidelines recommend temporary cessation of immunotherapy when an alternative AKI etiology cannot be identified, and initiation of corticosteroids at a dose of 0.5 to 1 mg/kg/day prednisone equivalents tapered over 4 to 6 weeks (88, 89, 113). For patients with sustained stage 1 AKI and those with stage 2 or 3 AKI, kidney biopsy is strongly recommended if there are no contraindications in order to determine the exact cause of AKI (**Figure 2**). Once the diagnosis of ICI-related AKI is confirmed, corticosteroids treatment should be initiated (**Table 3**). A study in 429 patients with ICI-related AKI demonstrated that starting corticosteroids within 14 days following diagnosis of ICI-related AKI was associated with higher likelihood of renal recovery (odds ratio 2.64; 95% CI 1.58-4.41) (35). Recently, Baker et al., demonstrated that mortality was lower in ICI-treated patients who developed ICI-related AKI as compared to those where the underlying etiology of AKI was unrelated to ICI therapy (112).

**Table 3:** Proposed management of patients with presumed immune checkpoint inhibitors-related acute kidney injury (after other causes have been ruled-out).

AKI stage^*^	Treatment
Stage 1	Consider holding ICI therapy and other potential nephrotoxins (PPI, NSAIDs, other cancer drugs) and after conservative management (e.g., IV fluids) reevaluate within one week; if available biomarkers (i.e., CRP and uRBP/Cr) and both are WNL** and clinical evaluation has low probability for ICI-AKI, consider resuming ICI therapy if creatinine improved back or close to baseline. If kidney function is not improved, manages as AKI stage 2/3.
Stage 2/3	Hold ICI therapy Strongly consider kidney biopsy to confirm diagnosis unless contraindications are present. Biopsy confirm ICI-AKI may start prednisone 0.8-1mg/kg/day (max. 60-80 mg/day) and taper as bellow ***, pulse-dose *i.v.* corticosteroids (e.g., methylprednisolone, 0.5-1 g/day) for 2 to 3 days usually for patients with stage 3 AKI, followed by oral prednisone; checking CBC, CMP, UA, CRP, proteinuria/Cr in a week and if stable/improving recheck in one month and at the end of taper (approximately 8 weeks total treatment time)

AKI, acute kidney injury; CBC, complete blood count; CMP, comprehensive metabolic panel; Cr, creatinine; CRP, C-reactive protein; SCr, serum creatinine; ICIs, immune checkpoint inhibitors; UA, urinalysis; uRBP, urine retinol‐binding protein; WBC, white blood cell; WNL, within normal limitation.

^*^ Stage 1, SCr >0.3 mg/dL or 1.5x baseline prior ICI; AKI stage 2, SCr >2x baseline prior ICI; AKI stage 3, SCr >3x baseline or SCr >4mg/dL prior ICI.

^**^ If biomarkers are available, check if patient is on corticosteroids as it can lower CRP.

^***^ Most cases of ICI-AKI are acute interstitial nephritis, taper 10 mg every week till reach 20 mg and if SCr is improving continue taper 5 mg every week until discontinued. (Recommended Pneumocystis jirovecii pneumonia (PJP) prophylaxis with pentamidine and atovaquone). Glomerular pathology may require specify treatment.

As previously described (7), immunosuppression with prednisone (starting dose 0.8-1.0 mg/kg and maximal dose 60-80 mg daily) for stage 1 to 2 AKI, can generally obtain excellent therapeutic efficacy. Pulse-dose intravenous corticosteroids (e.g., methylprednisolone, 0.5-1 g/day for 2 to 3 days) are usually recommended for hospitalized patients with stage 3 AKI, followed by treatment of oral prednisone. Oral prednisone treatment for 8 to 12 weeks is recommended similar to other drug-indued AKI (65); but the duration of prednisone taper should depend on therapeutic efficacy. A retrospective single-center pilot study examined a small number of patients with ICI-related AKI and found that a shorter course of corticosteroids tapered withing 3 weeks yielded similar kidney outcomes compared with a longer course tapered within 6 weeks (114). However, larger randomized clinical trials with predominantly biopsy-proven ICI-related AKI (e.g., AIN) should be performed to confirm these findings. It should be noted that corticosteroid therapy in ICI-related AKI may need to be longer in patients with higher chance of relapse. This could be partially caused by the long half-life of ICIs, which be as high as 27 days for pembrolizumab (39).

In addition, a study shows that corticosteroid dosages were higher in patients with a complete response compared to those with a partial response (23). Although it is hard to compare the corticosteroid outcomes in retrospective studies, this suggests that the initial dose of prednisone may play a key role in AKI recovery. There is also a subset of patients with ICI-related AIN that do not respond to corticosteroid treatment (28). In such patients who presented with corticosteroid refractory or dependent, immunosuppression regimen should be promptly adjusted by switching or adding other immunosuppressant drugs (i.e., azathioprine, cyclophosphamide, cyclosporine, infliximab, mycophenolate mofetil, or rituximab) (25, 88, 89, 115–117), but the data is limited so far. A case series study including 10 patients with refractory or relapsed ICI-related AKI shows that majority of patients (80%) obtained a long period complete or partial remission following treatment with infliximab (118). However, the median time of corticosteroid therapy in this study was 3.5 weeks only which may explain the relapse. Mycophenolate mofetil, which is commonly used as a steroid-sparing agent in other forms of AIN, may also be effective in ICI-related AKI that is steroid refractory (119, 120).

Some studies have suggested that the outcomes are poor in patients with ICI-related glomerular diseases (112). However, a systematic review study including 45 patients with biopsy-proven ICI-related glomerular disease shows that almost all patients received corticosteroids, and 73% experienced complete or partial recovery of AKI (61).

Immunosuppression therapy for IRAEs may decrease anti-tumor efficacy of ICIs and the overall outcomes (121–123). There is mixed data on whether corticosteroid use for IRAEs worsen cancer survival (121–123); some studies have indicated that corticosteroids may reduce survival (124). In patients with non-small cell lung cancer who were treated with PD-L1 inhibitors, cancer outcomes were worse in those with prednisone doses ≥10 mg (125, 126). Therefore, corticosteroids should be also used cautiously.

### Rechallenge

The ICIs are typically withheld until AKI recovers, and some patients with severe AKI that does not fully recover need permanently discontinuation of ICI. Nevertheless, ICIs may be the only effective treatment option in some cancers, and ICI rechallenge has to be considered in those who recover from an episode of ICI-related AKI (127) (**Figure 2**). Patients with severe kidney dysfunction before ICI administration and whose kidney function takes longer (>6 weeks) to recover from an episode of ICI-related AKI very likely developed permanent kidney dysfunction (39). Some studies show that the recurrence rate of ICI-related AKI ranges from 5.1% to 40% after ICI rechallenge (16, 18, 22, 24, 29, 128, 129). Recently, a large, multicenter study shows that of the 121 patients rechallenged, only 20 (16.5%) developed recurrent ICI-related AKI. All patients with recurrent ICI-related AKI held their ICI therapy, and 70% (14 out of 20) were treated with corticosteroids, and 60% (12 out of 20) achieved renal recovery (35). Notably, it has been shown that the incidence of recurrent ICI-related AKI is relatively low, but patients who recovery from ICI-related AKI may be at risk of developing other IRAEs after rechallenge with ICIs. In addition, patients who developed recurrent AKI after ICI rechallenge were often receiving a concomitant AIN-inducible drug such as PPI, NSAIDs, and antibiotics (23, 24, 130). Patients who were receiving a potential offending drug (PPI, NSAID, antibiotic) at the time they developed ICI-related AKI may be good candidates for rechallenge, as long as they can strictly avoid these agents during ICI rechallenge. Before initiating rechallenge therapy, a detailed discussion about the risks and benefits should be discussed between the multidisciplinary team of oncologists and nephrologist with the patient. Once rechallenge is started, it is important to closely monitor for AKI recurrence in order to early recognize and take prompt intervention strategies. In addition, further studies are also required to identify specific biomarkers and risk factors that can predict IRAEs during ICIs rechallenge.

## Conclusions

The incidence of ICI-related AKI is rising due to increasing use of ICIs in patients with cancer. Early and accurate diagnosis of ICI-related AKI is important to guide therapeutic strategies. The most commonly reported kidney lesion is AIN. Nevertheless, glomerular disease, acute tubular injury, and electrolyte abnormalities have also been described. The differential diagnosis of AKI in patients receiving ICIs is broad and due to the lack of sensitive clinicopathologic and laboratory findings, future experimental and clinical studies are urgently needed to identify noninvasive biomarkers that can be used to diagnose and predict ICI-related AIN. The underlying molecular mechanisms of AKI caused by ICIs should be further investigated and clarified. Patients with ICI-related AIN should temporarily withhold ICI therapy and start corticosteroid therapy, whereas pure ICI-related ATN does not require discontinuation of ICI therapy. For rechallenge of ICI after ICI-related AKI, future studies are needed to compare immunosuppression strategies and determine the optimal timing of ICI rechallenge. The risk factors and the long-term outcomes of ICI-related AKI need be better understood.

## Author contributions

All authors contributed to the research, development, and content of the manuscript. All authors contributed to the article and approved the submitted version.

## Funding

Author MS is supported by NIH R01 DK130839. Author SH is supported by National Institute of Health K08 DK118120 from the NIDDK and by Mayo CCaTS grant number UL1TR002377.

## Conflict of interest

MES has served as a scientific advisory board member to Mallinckrodt.

The remaining authors declare that the research was conducted in the absence of any commercial or financial relationships that could be construed as a potential conflict of interest.

## Publisher’s note

All claims expressed in this article are solely those of the authors and do not necessarily represent those of their affiliated organizations, or those of the publisher, the editors and the reviewers. Any product that may be evaluated in this article, or claim that may be made by its manufacturer, is not guaranteed or endorsed by the publisher.
